# Microbial Community Profiling of Human Saliva Using Shotgun Metagenomic Sequencing

**DOI:** 10.1371/journal.pone.0097699

**Published:** 2014-05-20

**Authors:** Nur A. Hasan, Brian A. Young, Angela T. Minard-Smith, Kelly Saeed, Huai Li, Esley M. Heizer, Nancy J. McMillan, Richard Isom, Abdul Shakur Abdullah, Daniel M. Bornman, Seth A. Faith, Seon Young Choi, Michael L. Dickens, Thomas A. Cebula, Rita R. Colwell

**Affiliations:** 1 CosmosID, College Park, Maryland, United States of America; 2 Maryland Pathogen Research Institute, University of Maryland, College Park, Maryland, United States of America; 3 Battelle, Columbus, Ohio, United States of America; 4 Department of Biology, Johns Hopkins University, Baltimore, Maryland, United States of America; 5 University of Maryland Institute for Advanced Computer Studies, University of Maryland, College Park, Maryland, United States of America; 6 Bloomberg School of Public Health, Johns Hopkins University, Baltimore, Maryland, United States of America; University of Hyderabad, , India

## Abstract

Human saliva is clinically informative of both oral and general health. Since next generation shotgun sequencing (NGS) is now widely used to identify and quantify bacteria, we investigated the bacterial flora of saliva microbiomes of two healthy volunteers and five datasets from the Human Microbiome Project, along with a control dataset containing short NGS reads from bacterial species representative of the bacterial flora of human saliva. GENIUS, a system designed to identify and quantify bacterial species using unassembled short NGS reads was used to identify the bacterial species comprising the microbiomes of the saliva samples and datasets. Results, achieved within minutes and at greater than 90% accuracy, showed more than 175 bacterial species comprised the bacterial flora of human saliva, including bacteria known to be commensal human flora but also *Haemophilus influenzae*, *Neisseria meningitidis*, *Streptococcus pneumoniae*, and Gamma proteobacteria. Basic Local Alignment Search Tool (BLASTn) analysis in parallel, reported *ca*. five times more species than those actually comprising the *in silico* sample. Both GENIUSand BLAST analyses of saliva samples identified major genera comprising the bacterial flora of saliva, but GENIUS provided a more precise description of species composition, identifying to strain in most cases and delivered results at least 10,000 times faster. Therefore, GENIUS offers a facile and accurate system for identification and quantification of bacterial species and/or strains in metagenomic samples.

## Introduction

The microbial flora of the human mouth has been extensively studied, providing an understanding of the role of bacterial species, not only in maintaining wellness, but also in dental caries and gingivitis[1], [2], [3]. The reductionist approach to understanding the microbiology of the human mouth that was followed in the early days of dental microbiology, in some ways inhibited achievement of full understanding of the oral flora as a microbial ecosystem. Recent research on the human microbiome has provided valuable information concerning a variety of processes, including interactions among microbial species in the human mouth. Next generation sequencing (NGS) permits an even more extensive characterization of the microbial ecology of the human body and has triggered an explosion in human microbiome discovery. As a result, the microbiome is now considered by some investigators to represent yet another organ of the human body, dictating health and well-being[4]. From the avalanche of data reported to date, spatio-temporal and host induced variations in microbiomes have been associated with a variety of human conditions, including colorectal carcinoma[5], [6], cardiovascular disease[7], inflammatory bowel disease[8], obesity [9], white blood cell cancer [10], and even psychiatric conditions[11].

The oral cavity is a major gateway for bacterial entry to the human body and a natural route for passage to respiratory and digestive tracts and, ultimately, the blood stream. Historically, microorganisms in the oral cavity were found to comprise a diverse and complex community [12], comprised of hundreds of individual bacterial species[13], [14]. Recent evidence shows that some bacterial species of the mouth microbiome are linked to oral disease, but are also important in the general health of an individual [12], [15], [16], [17], [18], [19]. To date, the oral microbiome has been linked to many diseases, namely alveolar osteitis and tonsillitis [12], [14], [20], [21], [22], [23], bacteremia [24], endocarditis [25], brain and liver abscesses [26], [27], stroke [28], diabetes [29], [30], pneumonia [31], and premature birth [32]. The mouth can also be considered an important site of genetic exchange among members of the bacterial flora, because of its high bacterial load and richness in species diversity, even where antibiotic resistant bacteria can become established through contact transmission [33]. The oral microbiome is not homogenous but is made up of subpopulations inhabiting microenvironments within the mouth [15]. A primary example is saliva, which contains a specific bacterial community that helps maintain homeostasis of the mouth ecosystem. Thus, it is not surprising that the oral or salivary microbiome has attracted increased attention as a potential diagnostic tool [23], [34], [35], [36]. Collection of saliva samples is simple and not invasive [15], [16], [37]. Saliva itself is clinically informative, containing many soluble biomarkers typically found in blood and urine [38], [39] that are useful in prognosis of several systemic and oral conditions. In fact, the uniqueness of individual oral commensal flora provides a forensic tool, contributing to the development of the new discipline microbial forensics [40], [41]. Because the salivary microbiome has diagnostic, epidemiological, and forensic value, we investigated the bacterial flora of saliva using direct whole genome shotgun (WGS) metagenomic sequencing, an unbiased metagenomic approach to determine the bacterial species and strain composition.

Historical microbiological studies employing conventional culture methods had shown that the human salivary microbiome is comprised of a complex assemblage of bacteria, viruses, fungi, and parasites, with less than half of the bacterial species cultured [12], [13], [14], [42]. 16S rRNA-based identification revealed extensive bacterial diversity, providing results more quickly than traditional culture [23]. However, 16S rRNA is a single gene-centric method, providing less resolution in differentiating closely related species. It also suffers from limitations imposed by non-uniform distribution of sequence dissimilarity among taxa, presence of multiple copies of the 16S rRNA gene [43], failure of target amplification of polymerase chain reaction (PCR) primers [44], and generation of chimeric sequences [45], [46].

The accuracy and robustness of any identification methods is dependent on the quality and breadth of the reference database [12]. The popular sequence alignment tool, BLAST [47] relies on the NCBI public database from which even NCBI removes sequences and genomes due to errors [48]. The known limitations of 16S rRNA sequencing for microbial identification has prompted investigators to use WGS sequencing for characterization and resolution of metagenomic communities [12], [36], The large amounts of data produced by WGS sequencing, however, present significant challenges in data analysis and interpretation [49]. There are many approaches that have been devised for analysis of WGS data, including alignment, assembly, binning, and gene prediction based methods [50], [51]. Read reference alignment or mapping performs reasonably well, but these are computationally very expensive [52]. Compositional binning tools, i.e. MEGAN [53] and MG-RAST [54], are also computationally expensive and do not resolve closely related taxa with the short reads as generated by Illumina and Life Technologies NGS platforms [50].

NGS has progressed today to being able to deliver highly accurate sequences economically and with fast turn-around. As a result, whole genome shotgun (WGS) metagenomic sequencing emerged as a powerful tool for studying the human microbiome [55]. At present, WGS metagenomic data comprise millions to billions of short reads, aiding necessary sequencing depth as needed as well as offering an unprecedented opportunity to identify individual species at or near strain level and determine their relative abundance. In this study, WGS metagenomics was employed, in combination with GENIUS algorithms, to identify and quantitate bacterial species comprising the salivary microbiome.

## Materials and Methods

### Sample Collection and DNA Isolation

Total DNA was collected from saliva samples provided by two anonymous healthy adult donors following the approved protocol of the Battelle Memorial Institute Internal Review Board. DNA was purified from saliva employing the Oragene-DNA isolation kit (DNA Genoteck, Kanata, ON, Canada), following the manufacturer’s recommended protocol.

### Next Generation Sequencing and Filtering

DNA samples for metagenomics were prepared for 150 bp and 100 bp single-end sequencing using the Illumina GAIIx and HiSeq 2000 instrument (Illumina, San Diego, CA), respectively. Numerically coded aliquots of approximately 0.5–1 µg DNA per sample were used to create sequencing libraries. First, genomic DNA was fragmented using a Covaris™ S220 Sonicator (Covaris, Inc., Woburn, MA) to approximately 300 base pairs (bp). Fragmented DNA was used to synthesize indexed sequencing libraries using the TruSeq DNA Sample Prep Kit V2 (Illumina, Inc., San Diego, CA), according to manufacturer’s recommended protocol. Cluster generation was performed on the cBOT using the TruSeq PE Cluster Kit v3 – cBot – HS (Illumina). Libraries were sequenced with an Illumina HiSeq 2000 at Nationwide Children’s Hospital (NCH) Biomedical Genomics Core (Columbus, Ohio) using the TruSeq SBS Kit v3 reagents (Illumina) for paired end sequencing with read lengths of 100 base pairs (bps) (200 cycles) and at CosmosID with an Illumina GAIIx for 150 base pairs (bps) single read using the TruSeq SBS Kit v5 reagents (Illumina). Primary analysis (image analysis and basecalling) were performed using HiSeq Control Software (HCS) version 1.5.15.1 and Real Time Analysis (RTA) version 1.13.48. Secondary Analysis (demultiplexing) was performed using Illumina CASAVA Software v1.6 Post processing of GAIIx reads was performed with RTA/SCS v1.9.35.0 and CASAVA 1.8.0 software. High throughput sequencing reads were quality filtered using the fastq_quality_filter program provided with the FASTX-Toolkit (http://hannonlab.cshl.edu/fastx_toolkit/index.html) (v. 0.0.13). Only those reads with a quality score ≥17 for at least 80% of the read length (i.e., probability of correct base call ∼98%) were retained. Ion Torrent (Life Technologies, NY) sequencing was also performed using amplicons specific to the V4 region of the 16S rRNA gene. Sequence reads are available under NCBI BioProject ID PRJNA231652.

### BLASTn Analysis

Sequence data were compared to the NCBI RefSeq database (v. May 19, 2012), but restricted to microbial gis, the NCBI 16S database (v. October 30, 2012), using BLASTn [47] (top hit only) (v. 2.2.25, National Library of Medicine, Bethesda, MD). Table 1 provides details of analyses carried out for each sample with data bases used. Resulting BLASTn hits were filtered to retain only those hits with percent identity ≥97%. An additional filter was applied to the BLASTn hit report to reduce false positives (i.e., reads whose corresponding taxonomic identifier (taxid) appeared ≤0.01% (1∶1000)). This was accomplished using a custom script. The Krona (v. 2.2) [56] program, ClassifyBLAST.pl, was also used within a custom script, to obtain a list of organisms identified with read counts associated with each taxon. Krona ImportBLAST.pl program was used to provide interactive visualization of identified bacterial species.

**Table 1:** Multi-platform BLASTn analysis of two salivary samples against various databases.

Sample	Sequencing Platform		
	Illumina HiSeq 2000	Illumina GAIIx	Ion Torrent (16S, V4)
	Reference Database used with BLASTn		
**VFD10-018**	Greengenes 16s^1^	Greengenes 16s^4^	Greengenes 16s^3^
	NCBI 16s	NCBI 16s^4^	NCBI 16s^3^
	NCBI RefSeq (microbial subset)^1^	NCBI RefSeq (microbial subset)^4^	NCBI RefSeq (microbial subset)^3^
**VFD12-006**	Greengenes 16s^1^	Greengenes 16s^2^	Greengenes 16s^3^
	NCBI 16s^1^	NCBI 16s^2^	NCBI 16s^3^
	NCBI RefSeq (microbial subset)^1^	NCBI RefSeq (microbial subset)^2^	NCBI RefSeq (microbial subset)^3^

^1^Illumina HiSeq2000 sequencing carried out at Nationwide Children’s Hospital, Columbus, OH.

^2^Illumina GAIIx sequencing carried out at CosmosID.

^3^Ion Torrent sequencing performed by SeqWright, Inc., Houston, TX.

4 Illumina GAIIx sequencing performed at The Ohio State University, Columbus, OH.

### GENIUS Analysis

Raw unassembled WGS short reads generated by the Illumina GAIIx and HiSeq 2000 platforms were analyzed using GENIUS software package for rapid identification of bacterial species and relative abundance. GENIUS creates sample libraries from unassembled short WGS reads using two algorithms, 5VCE and NmerCE, and utilizes GeneBook® reference libraries derived from curated genomic databases to assign taxonomic membership of sample libraries, employing probabilistic matching. Identification is achieved at species, sub-species, and/or strain level, depending on adequate representation of relevant reference genomes in the GeneBook® libraries

### *In silico* Metagenome Construction

A synthetic metagenome was created comprising a total of 5.5 M reads from ten bacterial species and the human genome (Table 2). The reads, each 100 nucleotides in length, were created using a custom R script from each of the ten bacterial genome and the human chromosome 21 using Illumina sequencing error model.

**Table 2:** Species composition and simulation statistics of the synthetic metagenomic dataset.

Simulations Statistics				BLAST (RefSeq_genomic)
Species	Genome Coverage	Number of Reads	Relative Abundance	Number of Reads
*Homo sapiens*	0.08	4034394	72.81	3349
*Rothia dentocariosa* ATCC 17931	47.21	537919	9.71	554
*Prevotella melaninogenica* ATCC 25845	52.69	430335	7.77	440
*Fusobacterium nucleatum subsp. nucleatum* ATCC 25586	5.44	53791	0.97	58
*Streptococcus oralis* Uo5	12.08	107583	1.94	106
*Streptococcus mitis* B6	11.02	107583	1.94	88
*Veillonella parvula* DSM 2008	11.1	107583	1.94	83
*Peptostreptococcus stomatis* DSM 17678	0.99	46261	0.83	NA
*Peptostreptococcus anaerobius* 653-L	0.94	46261	0.83	NA
*Porphyromonas gingivalis* W83	0.84	46261	0.83	46
*Mycoplasma pneumoniae* FH	1.21	23130	0.42	23

The right most column represents number of reads from the sample that BLAST was able to assign to species. NA: Not Assigned due to lack of RefSeq entries.

## Results and Discussion

Description of the human microbiome has been made possible by NGS with its significant reduction in cost and improvement in throughput. Metagenomics, as a result, is moving from a16S rRNA gene-centric approach to WGS metagenomic approach. To date, 16S rRNA gene sequencing has been used to identify major taxa and explore the microbial diversity of the human salivary microbiome [23], [35], [57] linking composition of the microbiome with oral health and/or systemic disease. In this study, WGS metagenomics was used, along with several bioinformatics analysis methods e.g., BLASTn, mapping, and GENIUS, to determine relative performances of taxonomic assignment, and identification of community composition and structure, thereby achieving improved understanding of the human salivary microbiome.

GENIUS 5VCE algorithm was employed to determine the bacterial species composition of a human saliva sample, VFD10-018, sequenced by the Illumina GAIIx (150 bp, ∼22 M reads) platform. A total of 26 bacterial genera and 58 species were identified with majority of genera and species previously identified as members of the human salivary and/or oral microbiome (http://www.homd.org/index.php, Fig. S1). BLASTn (microbial subset) and short read mapping (CLC genomic workbench, using same genome database as GENIUS 5VCE) identified 45 and 102, and 67 and 108, bacterial genera and species in this data set respectively, indicating a much larger microbial community compared to that identified by GENIUS 5VCE (Fig. S1). A global 16S metagenic survey of saliva samples collected from 120 healthy individuals in 12 geographically different locations reported that an individual salivary microbiome typically contains six to 30 bacterial genera [41], an observation in agreement with GENIUS 5VCE identification. Dominant genera identified by GENIUS 5VCE (*Streptococcus,Prevotella, Veillonella*, *Mycoplasma, Rothia*, *Haemophilus, Fusobacterium etc.)* were also in agreement with genera identified in the global survey [41]. It is concluded that bacterial taxa identified by BLASTn and short read mapping produces an overestimation of diversity. GENIUS 5VCE performed favorably in both speed, at least 10,000X faster than BLAST, and accuracy in identifying the most likely bacterial community of this dataset with significantly reduced false prediction.

**Figure 1:**
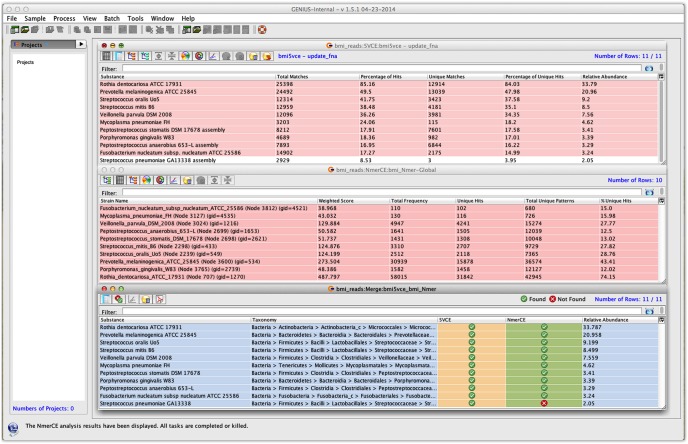
Screenshot of GENIUS client software displaying tabular output of *in silico* metagenomic data. Left panel indicates projects loaded to this graphical user interface. Three table views to the rightmost panel represent output of 5VCE (a)NmerCE (b) and merged output from both 5VCE and NmerCE (c) algorithms, respectively.

As the actual microbial composition of the saliva samples from the volunteers was unknown, only comparative analysis between orthogonal methods was possible. Therefore, an *in silico* sample (Table 2) containing a composite of 5.5 M reads from ten bacterial species and the human genome was prepared to measure accuracy of the metagenomic analysis. Results show that GENIUS accurately identified bacterial species composition with a negligible computation time (2 minutes for NmerCE and 29 minutes for 5VCE) (Figs. 1 and S2). Despite the fact that the number of sequencing reads for each of the genomes comprising the test set was small, the GENIUS algorithms identified the bacterial species with appropriate strain designation. Identification obtained using GENIUS 5VCE and NmerCE algorithms were in agreement, with one possible false positive identification (*Streptococcus pneumoniae*) by 5VCE. When statistical analysis was carried out, which is a built in function in GENIUS 5VCE algorithm, to provide point estimates for genome coverage and detection confidence limits using random k-mers for each of the identified species, *S. pneumoniae* had the lowest confidence interval (Fig. S2). Furthermore, considering the marginal coverage of strain specific attributes (only 1.6% of unique identifiers), error rate of the sequencing platform, and abundant presence of other *Streptococcus* species in the dataset, this call, at best, would have been tallied as dubious. In contrast, results of BLASTn analysis reported 48 species, even though the sample contained only ten bacterial species, confirming what has been suspected by other investigators as over-estimation of diversity by BLASTn analysis. In contrast, GENIUS was efficient in filtering out false positive signals caused by high genomic similarity among closely related genera and species. Briefly, GENIUS was successful in identifying species of the saliva microbial community, even when only limited sequencing data were available, accomplishing identification that required only minimum computational time. Precise identification was achieved in most cases even with only a small number of reads, i.e., fractional coverage (<1%) of the genome (i.e.,*Peptostreptococcus stomatis, Peptostreptococcus anaerobius*, and *Porphyromonas gingivalis*) were available and also when the target pathogen (i.e., *Mycoplasma pneumoniae)* was present at very low concentration (<1%).

**Figure 2:**
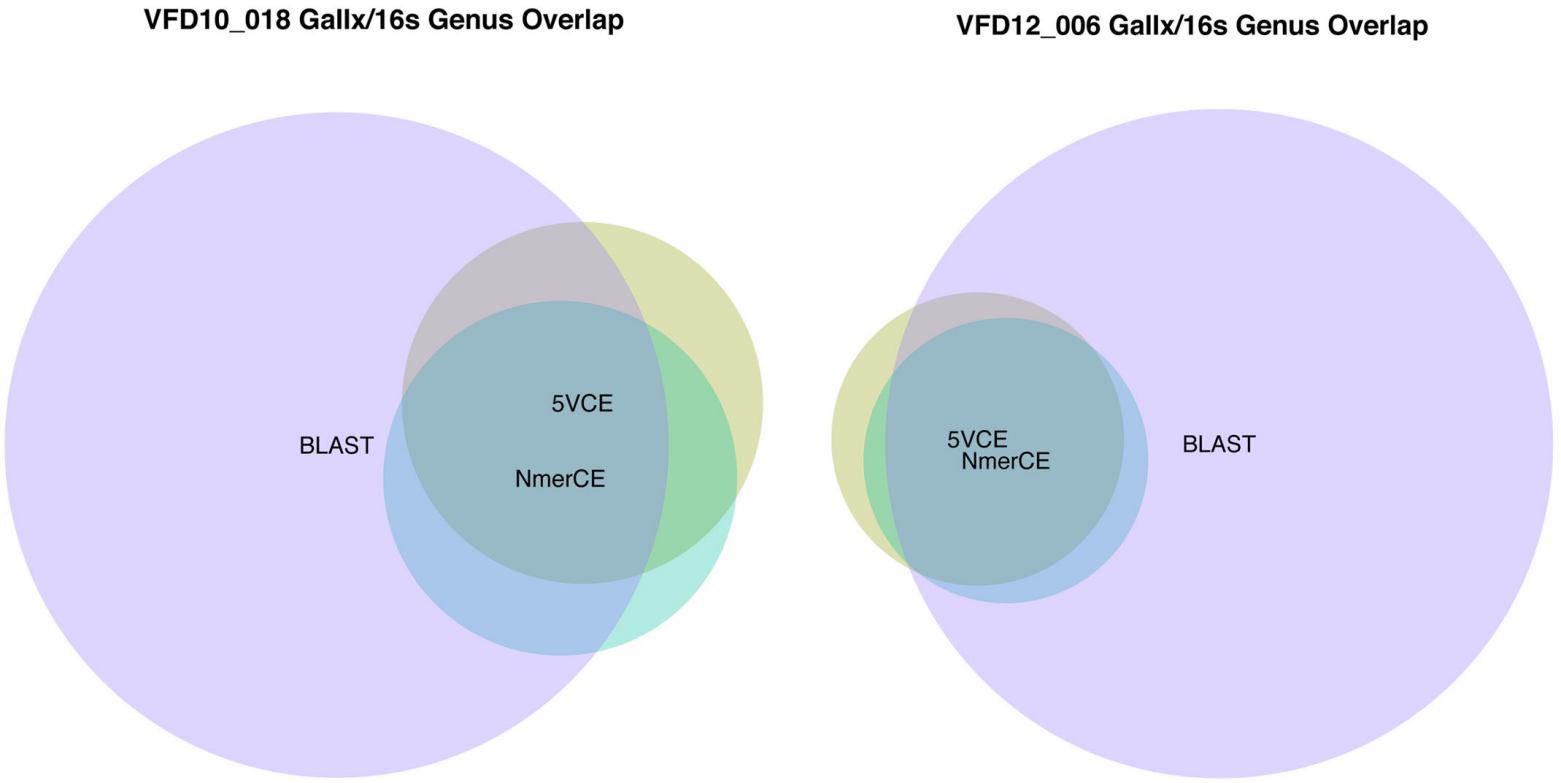
Genus overlap for sample VFD10-018 (a) and VFD12-006 (b) estimated by 16S sequencing/NCBI 16S BLAST and GAIIx sequencing/5VCE-NmerCE.

For comparative purposes, 16S rRNA (V4 region)sequencing was carried out using Ion Torrent PGM for salivary samples VFD10 and VFD12 and the sequence data were analyzed by BLAST, using the NCBI 16S ribosomal database (v. 10/30/2012) to enable direct comparison of bacterial communities inferred by both 16S metagenic and WGS metagenomics analysis. Comparison of the accuracy of identification between the two methods was assessed by the number of overlapping genera, since extrapolation of 16S data beyond genus is very limited [43], [58], [59]. Saliva microbiomes VFD10 and VFD12 showed high concordance (∼80%), with respect to genera identified by GENIUS algorithms 5VCE and NmerCE (Fig. 2). Concordance was shared, to a large extent, with 88 genera identified by BLAST-16S (Fig. 2a). GENIUS algorithms 5VCE and NmerCE identified 27 and 26 genera, respectively, in VFD10. Of the 21 genera identified by both algorithms, 17 were also identified by BLAST-16S. The six additional genera identified by5VCE (n = 2) and NmerCE (n = 4) were also identified by BLAST-16S. However, five genera were identified by GENIUS, either by5VCE (n = 4) or NmerCE (n = 1) that were not detected by BLAST-16S. Relative abundance (≥1%)of genera determined by GENIUS algorithms and byBLAST-16S (Table S1) were in agreement, with respect to dominant genera, with26 genera comprising 97–99% of the bacterial community.

Results for saliva sample VFD12 are shown in Fig. 2b, with comparison of relative abundance showing 22 genera accounting for 98–99% of the bacterial community (Table S2), providing very little evidence for the large number of genera (n = 82) identified by BLAST-16S. In fact, the global survey of saliva samples [41]reported individual salivary microbiomes contained six to 30 bacterial genera, reinforcing overestimation of diversity by 16S analysis. Based on the literature,16S rRNA sequencing can be biased by unequal amplification of 16S rRNA genes [46] and by taxon-specific biases arising from the primer set used [60]. Generation of chimeric sequences also can skew and inflate diversity estimates significantly [45], [46]. Chimera formation is most pronounced when 16S rDNA amplicons are present in low amount, making identification of minor species suspect without supporting data [61].

**Figure 3:**
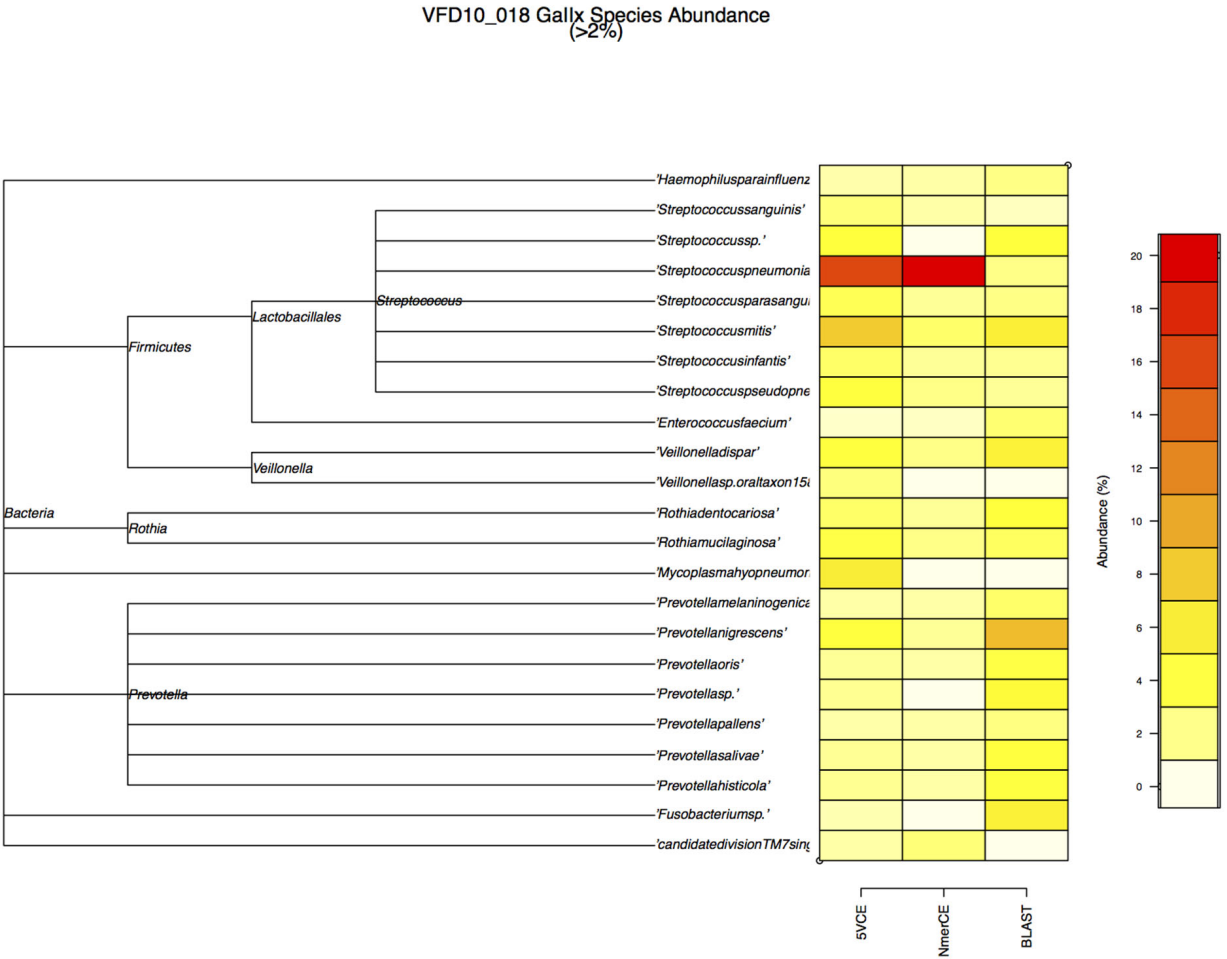
Relative abundance of species in VFD10-018 estimated by GAIIx sequencing and BLAST (microbial reference database), 5VCE, and NmerCE algorithms.

**Figure 4:**
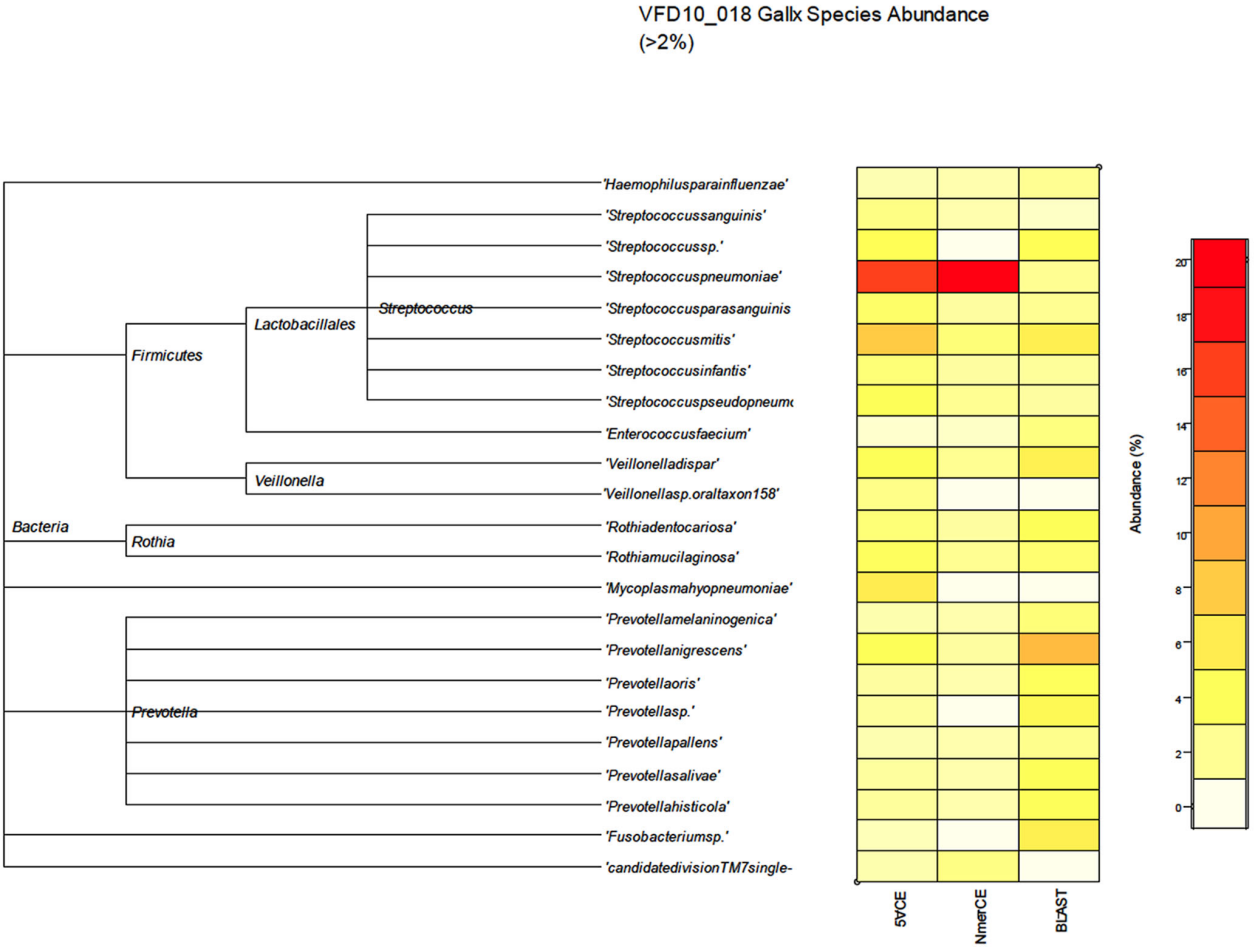
Relative abundance of species in VFD12-006 estimated by GAIIx sequencing and BLAST (microbial reference database), 5VCE, and NmerCE algorithms.

WGS metagenomics sequencing reads (GAIIx) were compared with the NCBI RefSeq database (microbial subset, v. 05/19/2012), using BLASTn for comparison of species identified by GENIUS algorithms and BLASTn. Fig. 3 shows estimated relative abundance of species at ≥2% for saliva sample VFD10, determined by both GENIUS and BLASTn analyses. A phylogenetic tree of 23 species (depicted as shaded squares) in Fig. 4 shows relative abundance estimated by each method. Most prevalent were *Streptococcus* and *Prevotella*. Similar results were obtained for saliva sample VFD12 (Fig. 4).

A vexing problem in metagenomics is sample diversity, that is, whether sequencing captures the total diversity of a given sample. Libraries generated for saliva sample VFD10 were sequenced using all eight lanes of a GAIIx flow cell, with 18 to 24 million reads (average ∼22 million) generated per lane (Fig. S3). GENIUS analysis, with respect to number of species identified, showed good concordance in both identification and relative abundance of bacterial species, for all eight lanes (Fig. S3). Thus, use of an entire flow cell for a single sample library did not influence the diversity estimate.

Illumina HiSeq 2000 allows *ca*.180 million reads per lane, with TruSeq v3 chemistry, compared to *ca*. 40 million reads per lane, with GA v5 chemistry and the GAIIx instrument. When the larger number of reads generated by HiSeq (66–75 million) was analyzed, the number of species identified and percent of each species increased for both saliva samples VFD10 and VFD12, indicating that a larger number of HiSeq reads will contribute breadth and depth in genome coverage (Fig. S4). These results suggest that identification of bacterial species, particularly those present in low number, can be improved with a larger number of reads, especially in the case of samples containing large a mounts of human DNA. Analysis of saliva sample VFD10, using GAIIx sequence data, showed approximately 97% of the sequenced reads was from human DNA. Since background human DNA can range from less than 1% in stool samples to greater than 99% in nasal and vaginal samples [62], the effect of having an increased number of reads to capture species diversity will vary according to host DNA content, as well as complexity of the bacterial population. Improved sampling, extraction, and library construction methods, therefore, should be considered for maximum coverage of species diversity.

GENIUS was used to analyze salivary datasets from the Human Microbiome Project (HMP) (http://hmpdacc.org/HMASM/). Five human salivary microbiomes were analyzed and nine major phyla were identified by GENIUS 5VCE with Firmicutes, Bacteroidete, Actinobacteria, and Proteobacteria most abundant, Fusobacteria and TM7moderately abundant, and Spirochaetes, Synergistetes, and Tenericutes least abundant. Sixty seven bacterial genera belonging to nine phyla were identified, with eleven genera, *Streptococcus, Prevotella, Veillonella, Neisseria, Haemophilus, Campylobacter, Fusobacterium, Rothia*, *Mycoplasma,Actinomyces,and Aggregatibacter* comprising ∼90% of the bacterial community. Relative abundance estimates of phyla and genera varied (Fig. S5). Overall abundance and distribution of phyla and genera were in agreement with results of studies of human saliva reported by other investigators [34], [63], [64]. Interestingly, ***Streptococcus*** was observed to be prevalent in most of the HMP datasets, whereas *Prevotella* predominated in the saliva samples, VFD12 and SRS014692, analyzed in this study (Fig. S5). That is, a readily distinguishable abundance profile of ***Prevotella*** species and strains was observed in saliva samples VFD10, VFD12, and SRS014692 (Fig.S6). Greater abundance of ***Prevotella*** in caries-active, **compared to** healthy, individuals has been reported with caries-active individuals often carrying a mixture of *Prevotella* species different from normal healthy individuals [65].

GENIUS identified more than 175 bacterial species, including bacteria commensal to the human salivary microbiome ([65],HOMD, www.homd.org) but also others not usually found in the saliva flora of healthy individuals, including *Haemophilus influenza*e, *Neisseria meningitidis*, *Streptococcus pneumoniae* and Gammaproteobacteria. Several bacterial species, including ***Aggregatibacter actinomycetemcomitans, Porphyromonas gingivalis,*** *Treponema denticola, Fusobacterium nucleatum, Campylobacter rectus, Parvimonas micra, Eikenella corrodens, Prevotella melaninogenica, Prevotella nigrescens, Eubacterium saburreum*, and *Eubacterium yurii,* associated with periodontitis [12], [66], [67], [68], were also identified in varying abundance and distribution (Fig. S7). Although presence of these bacterial species may indicate disease, i.e., periodontitis, it has been shown that periodontitis can be attributed to genetic factors [69]. Therefore, diagnosis of periodontitis cannot yet be made by bacteria present in saliva.

**Figure 5:**
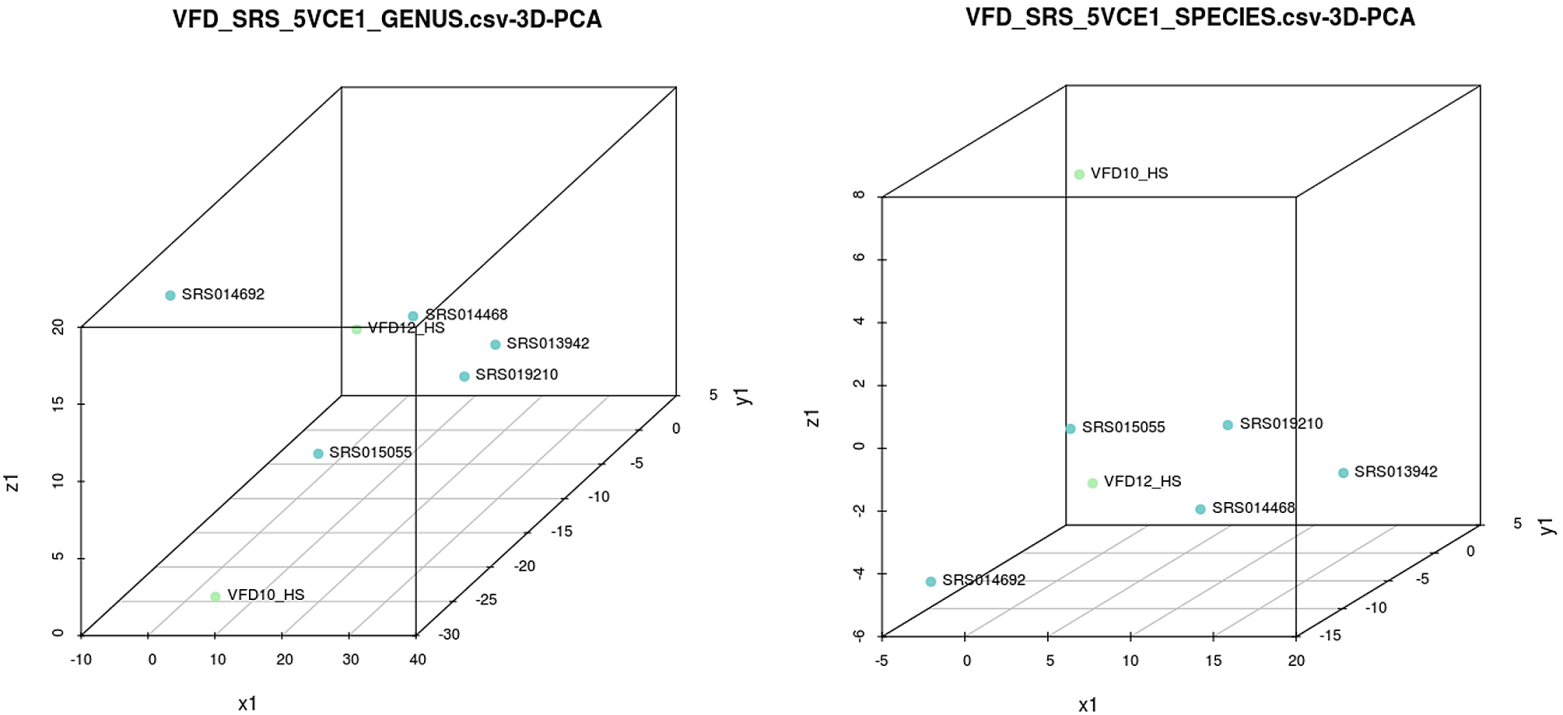
Principal component analysis of data for seven saliva samples analyzed by GENIUS.

**Figure 6:**
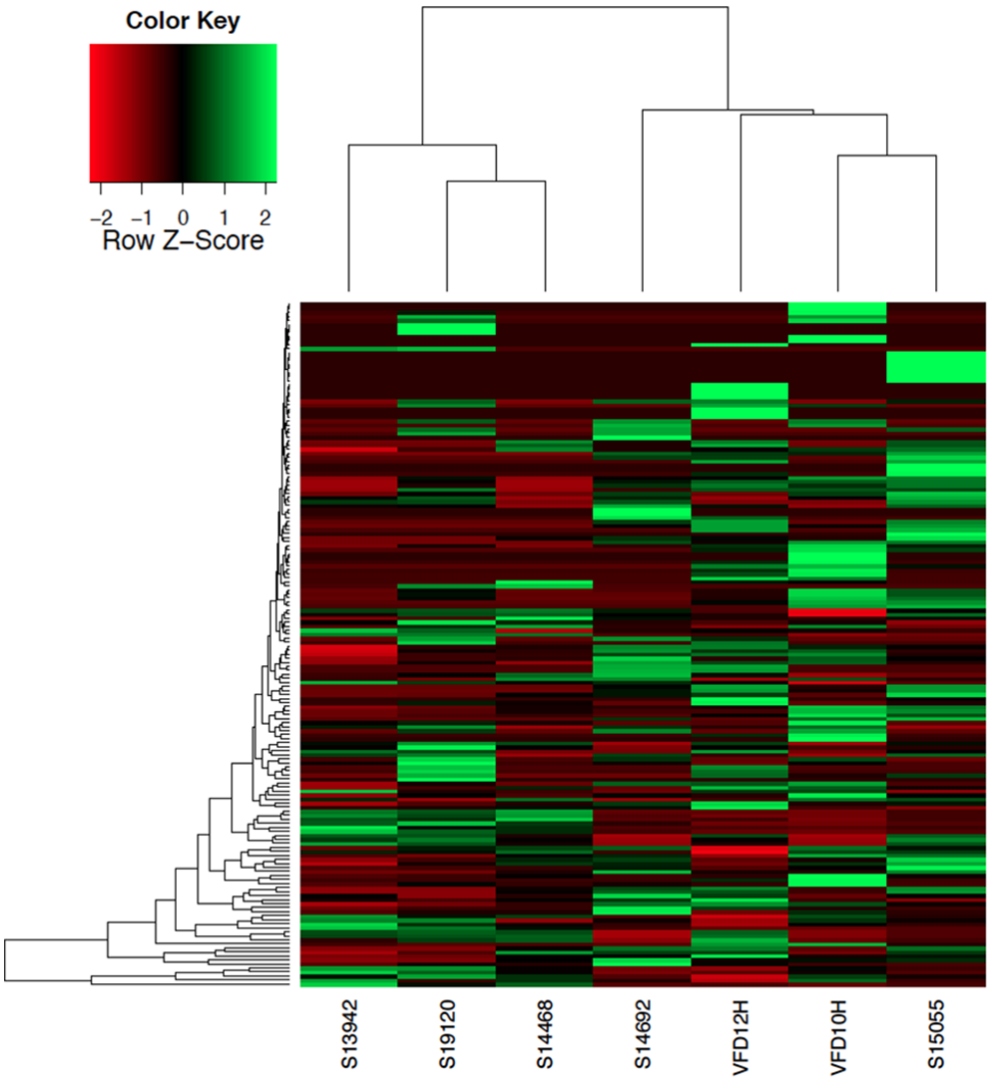
Double hierarchical dendrogram showing bacterial distribution at the species level for seven saliva samples. The relative values for bacterial species are depicted by color intensity, with legend indicated at the top of the figure.

Principal component analysis (PCA) of the five HMP saliva samples and the two saliva samples sequenced in this study showed the bacterial species composition comprised two major clusters (Fig. 5). Four saliva samples, VFD10, VFD12, SRS015055, and SRS014692, clustered separately from SRS09210, SRS013942, and SRS014468. Distinction between clusters, as well as variation within each population, was apparent from a double hierarchical dendrogram showing abundance and distribution of the bacterial species (Fig. 6). Even though two major clusters were observed, clearly diversity and species assemblage of the saliva samples were not identical. Such differences reflect diet, hygiene, and/or family and culture, all of which influence the oral microbiome. Centroid classification [70] within the two major groupings showed four genera, *Haemophilus*, *Streptococcus, Neisseria,* and *Aggregatibacter*, were over-represented in cluster B (Fig. S8). Over representation of *Haemophilus* species, i.e., *H. influenza*e, *H. parainfluenza*e, and *Aggregatibacter aphrophilus*(née, *Haemophilus aphrophilus*)in cluster B is particularly interesting, since these bacterial species have been shown to be associated with *Haemophilus* endocarditis [71], [72]. Although *Haemophilus* species can cause adult endocarditis (0.8–1.3%) [73], the presence of a significant number of each of these species represent a skewing from healthy human saliva.

## Conclusion

WGS metagenomics applied to the human microbiome has provided useful information applicable to public health and personalized medicine, especially as high-throughput ultra-deep sequencing approaches real time and becomes cost effective. However, post sequencing processing and analysis of data generated by WGS metagenomics are extremely challenging. While traditional BLAST analysis is hindered by factors like time for analysis, low resolution, and large computational requirements, a marker gene approach will speed detection, but sacrifices resolution. Genome mapping and reconstruction ensure precise identification but takes a long ‘time to identification’ and require powerful computational infrastructures and skilled manpower. In this study, GENIUS algorithms and WGS metagenomic data were used to identify the bacterial community composition of human saliva. Compared to16S metagenic sequencing and analysis, WGS metagenomics provided greater accuracy, both in identification and quantitation of bacterial species and less biased estimate of diversity, when GENIUS algorithms were used. Superior speed, accuracy, and precision in identification were achieved compared to 16S which significantly overestimated diversity. GENIUS algorithms provide high specificity and accurate identification of species, even those present in low abundance and with fractional genome coverage. WGS metagenomics employing GENIUS algorithms is proposed as method of choice for rapid, accurate, and user friendly bacterial identification and metagenomics.

In this study, it has been demonstrated that WGS metagenomics provides a practical approach in answering questions about the human salivary microbiome. Therefore, metagenomic analysis of clinical samples, not only of the salivary microbiome, but also other microbial flora, in general, offers greater power of decision making, precision, and speed compared to traditional methods. The typical “time to answer” for culture-based methods requires weeks for completion, whereas sequencing approaches can reduce the timeline to a few days. For WGS metagenomics for microbial detection and identification, laboratory protocols require a fraction of time compared to culture-based methods, especially since culturing is not required prior to library construction. The time required for a sample being processed and sequenced is approximately two days if MiSeq, 454 GS junior, Ion Torrent PGM, NextSeq or HiSeq X platforms are used for sequencing throughput. However, this timeline could be reduced through automation and will soon be less than a day. The main difference is actually the data analysis, when GENIUS is used, it takes only half an hour or less for analysis of metagenomic data derived from any routine clinical samples and does not require time-consuming alignment or mapping.

The cost of NGS has reduced dramatically and continues to decrease. It is clear that sequencing is not yet the least expensive method, but considering the amount of information obtained from NGS and the depth of resolution of the analyses, it is proving to be more cost-effective because of the greater breadth and depth of information provided compared to traditional methods involving culture and other bioassays, with battery of tests and reagents required. Metagenomics also provides opportunity to interrogate the same dataset against multiple databases (i.e, GeneBook libraries) for detection of bacteria, viruses and their virulence factors and/or antibiotic resistance in a single assay. It is concluded that application of GENIUS and GeneBook libraries can be utilized effectively for wider application in the clinical laboratory.

## Supporting Information

Figure S1 Comparative analysis of human saliva sample VFD10 sequenced by Illumina GAIIx using GENIUS 5VCE, BLAST (NCBI, microbial subset) and short read mapping.
(TIFF)

Figure S2 Statistical analysis of confidence interval by GENIUS and visualization of the metagenomic community using the Krona visualization tool.
(TIFF)

Figure S3 GENIUS 5VCE prediction of species relative abundance in eight lanes of an Illumina GAIIx flowcell. The smaller chart to the upper right corner represents the number of reads generated per lane.
(TIFF)

Figure S4 GENIUS 5VCE prediction of percent of total hits for identified bacterial species in VFD10 and VFD12, sequenced by both GAIIx and HiSeq 2000. The smaller chart to the upper right corner shows number of reads generated for two samples by GAIIx and HiSeq 2000.
(TIFF)

Figure S5 Abundance of bacterial phyla and genera identified in the salivary microbiome by GENIUS.
(TIFF)

Figure S6 Distribution and abundance of different *Prevotella* spp. and strains in salivary microbiomes.
(TIFF)

Figure S7 Occurrence and relative abundance of bacterial species associated with periodontal disease.
(TIFF)

Figure S8 The centroid classification analysis of cluster A and B salivary samples. The top ranking markers can differentiate cluster B (green) and cluster A (red) by the centroid scores. Species and genera with nonzero components in each class are almost mutually exclusive.
(TIFF)

Table S1 Comparison of relative abundance of genera in VFD10-018 using different methods.
(DOCX)

Table S2 Comparison of relative abundance of genera in VFD12-006using different methods.
(DOCX)
