# Correction: Microbial Community Profiling of Human Saliva Using Shotgun Metagenomic Sequencing

**DOI:** 10.1371/journal.pone.0106124

**Published:** 2014-08-18

**Authors:** The PLOS ONE Staff

Figure 4 is an accidental duplication of Figure 3. Please see the correct Figure 4 here.

**Figure 4:**
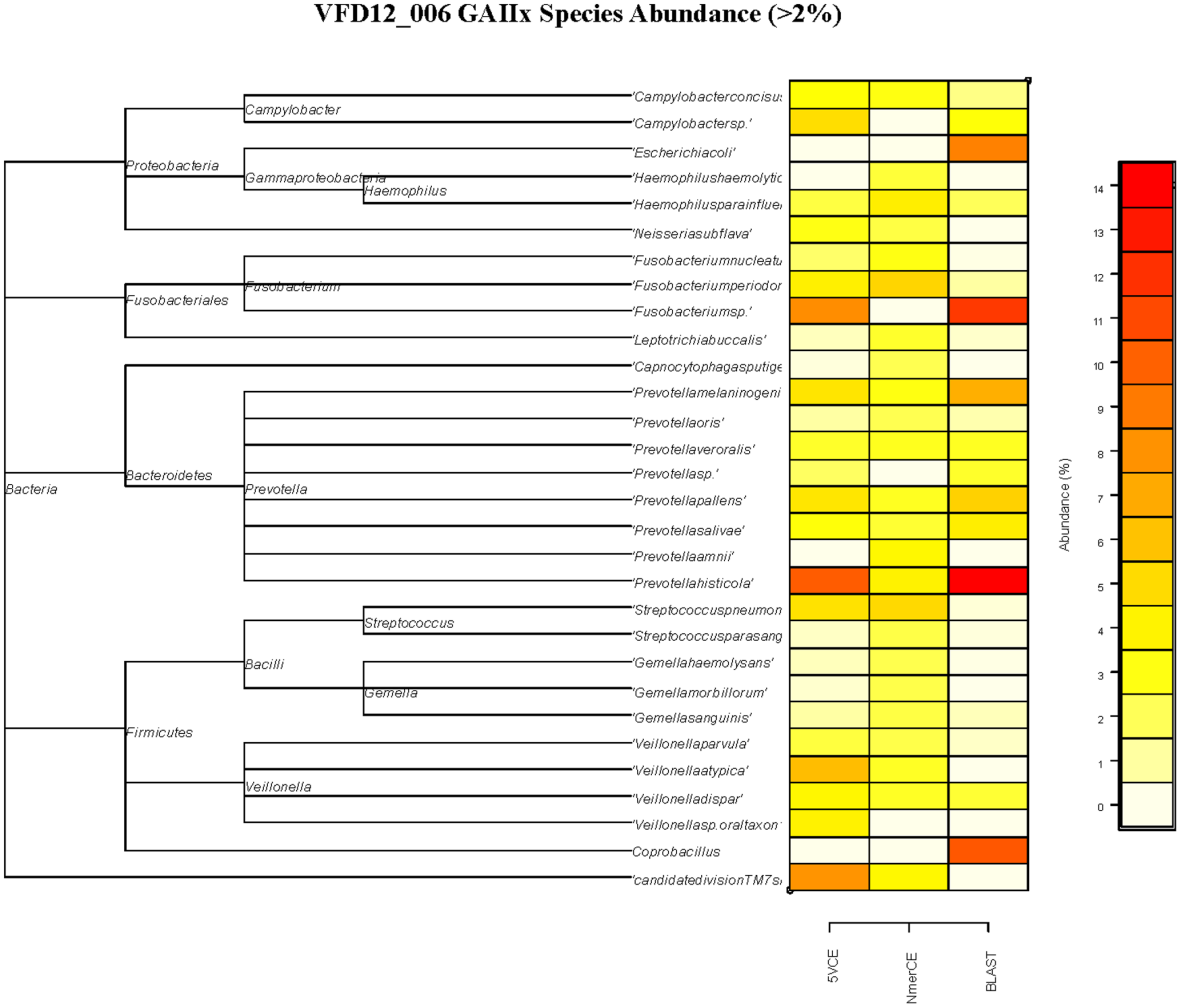
Relative abundance of species in VFD12-006 estimated by GAIIx sequencing and BLAST (microbial reference database), 5VCE, and NmerCE algorithms.
