# Supplementary figures and images for: Microbial Community Profiling of Human Saliva Using Shotgun Metagenomic Sequencing
Source: PLoS One. 2014 May 20;9(5):e97699. doi: 10.1371/journal.pone.0097699 (PMC4028220; doi:10.1371/journal.pone.0097699)

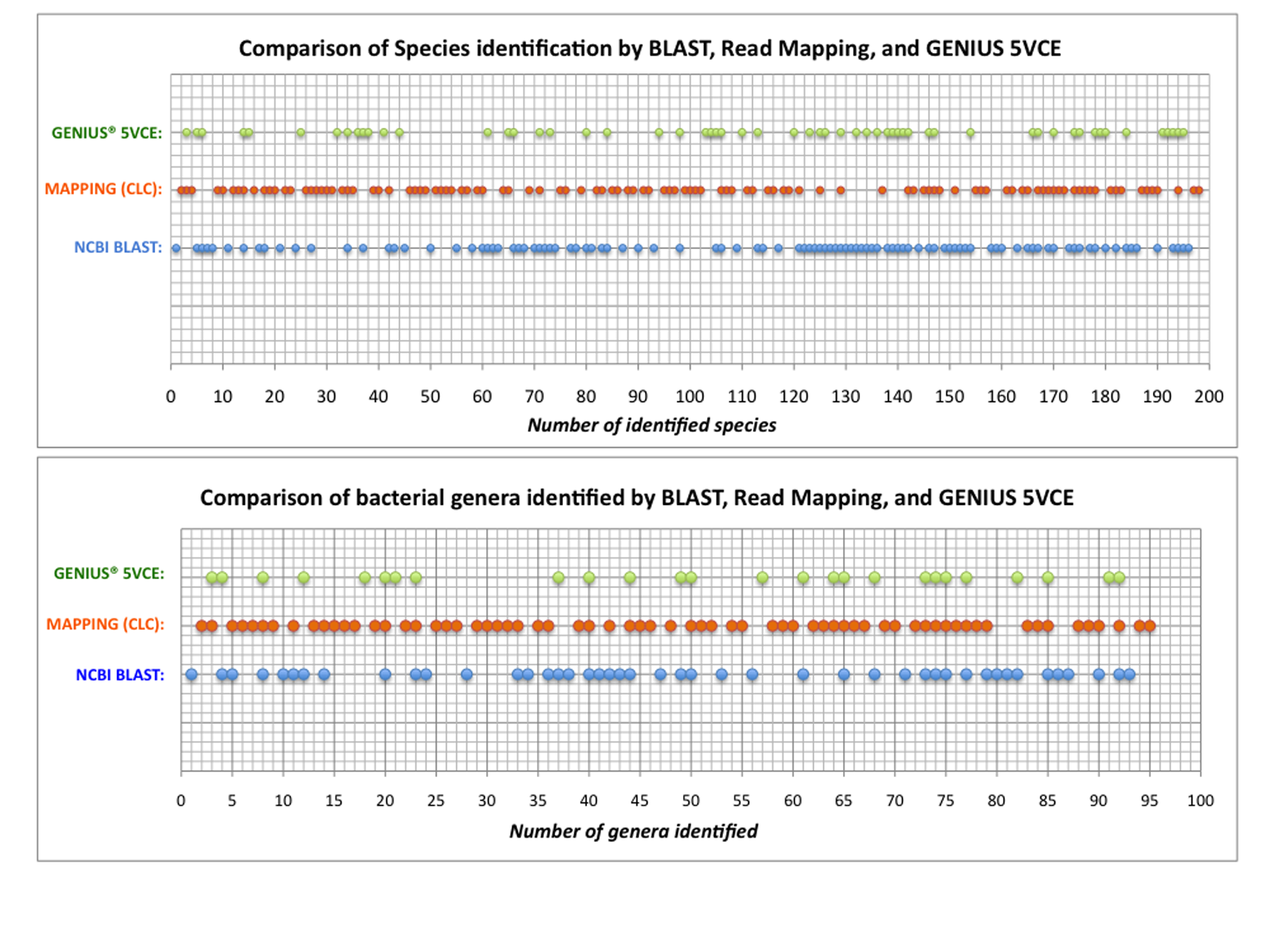

Supplement: Figure S1 — Comparative analysis of human saliva sample VFD10 sequenced by Illumina GAIIx using GENIUS 5VCE, BLAST (NCBI, microbial subset) and short read mapping. (TIFF) [file pone.0097699.s001.tiff]

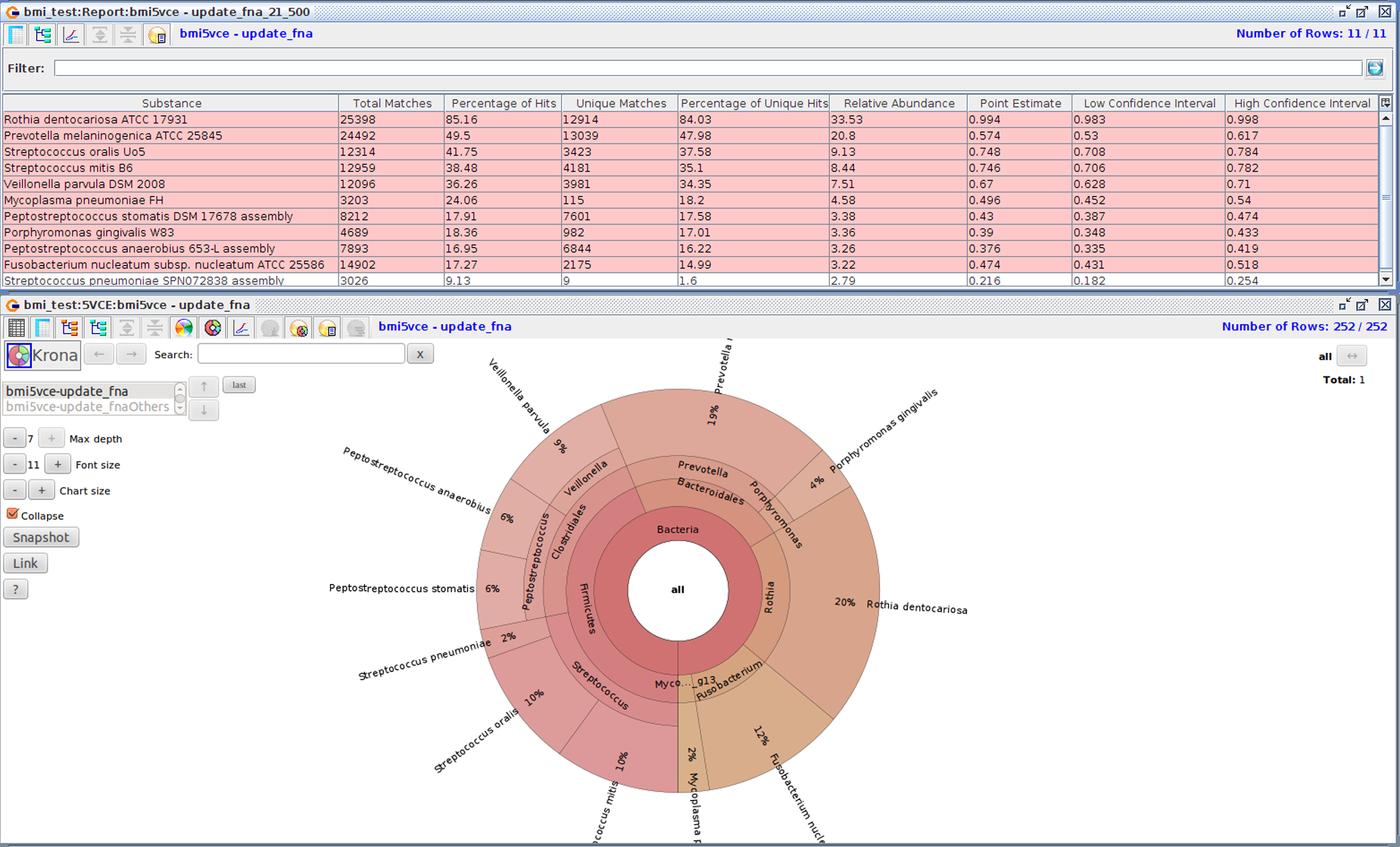

Supplement: Figure S2 — Statistical analysis of confidence interval by GENIUS and visualization of the metagenomic community using the Krona visualization tool. (TIFF) [file pone.0097699.s002.tiff]

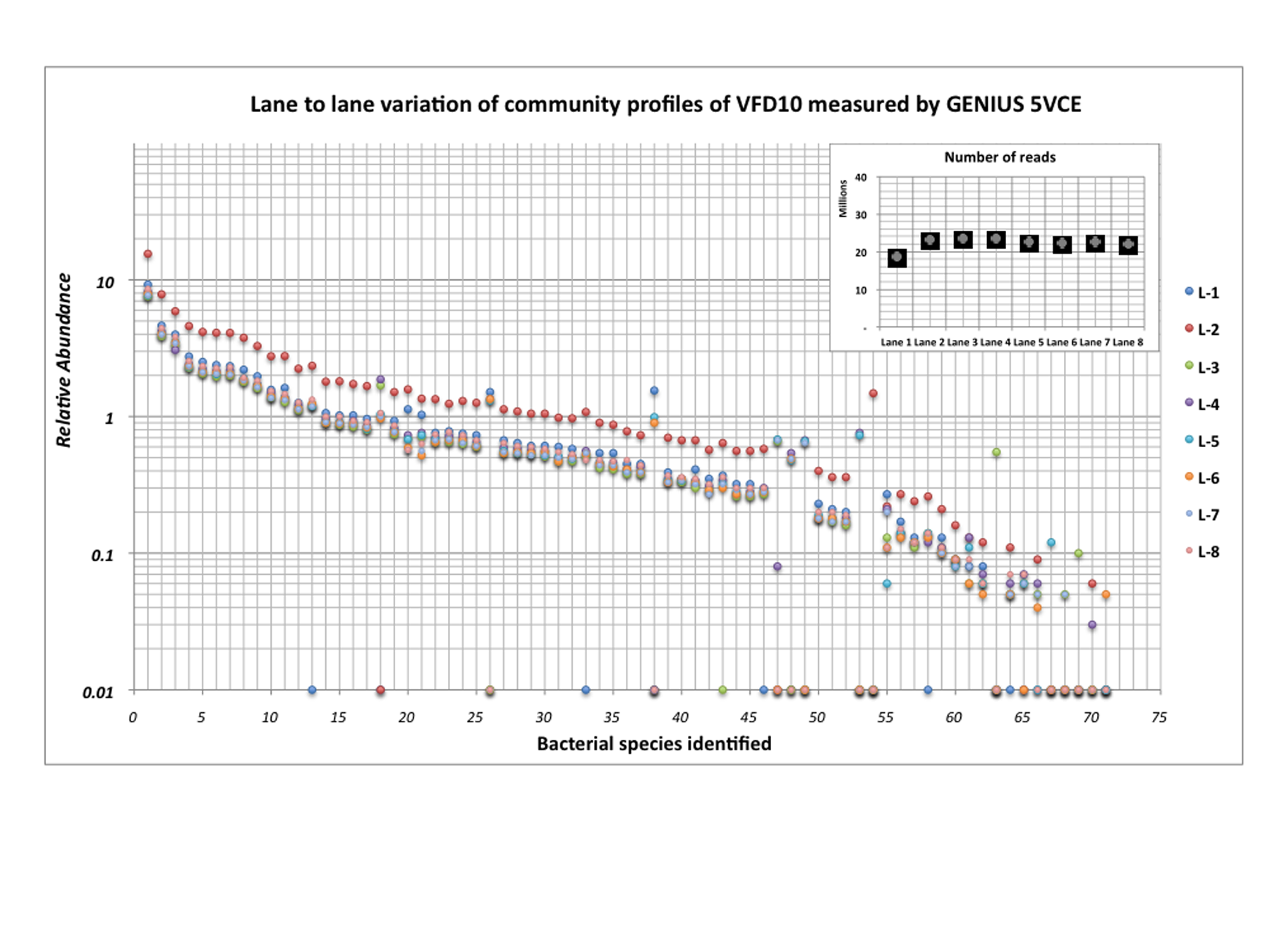

Supplement: Figure S3 — GENIUS 5VCE prediction of species relative abundance in eight lanes of an Illumina GAIIx flowcell. The smaller chart to the upper right corner represents the number of reads generated per lane. (TIFF) [file pone.0097699.s003.tiff]

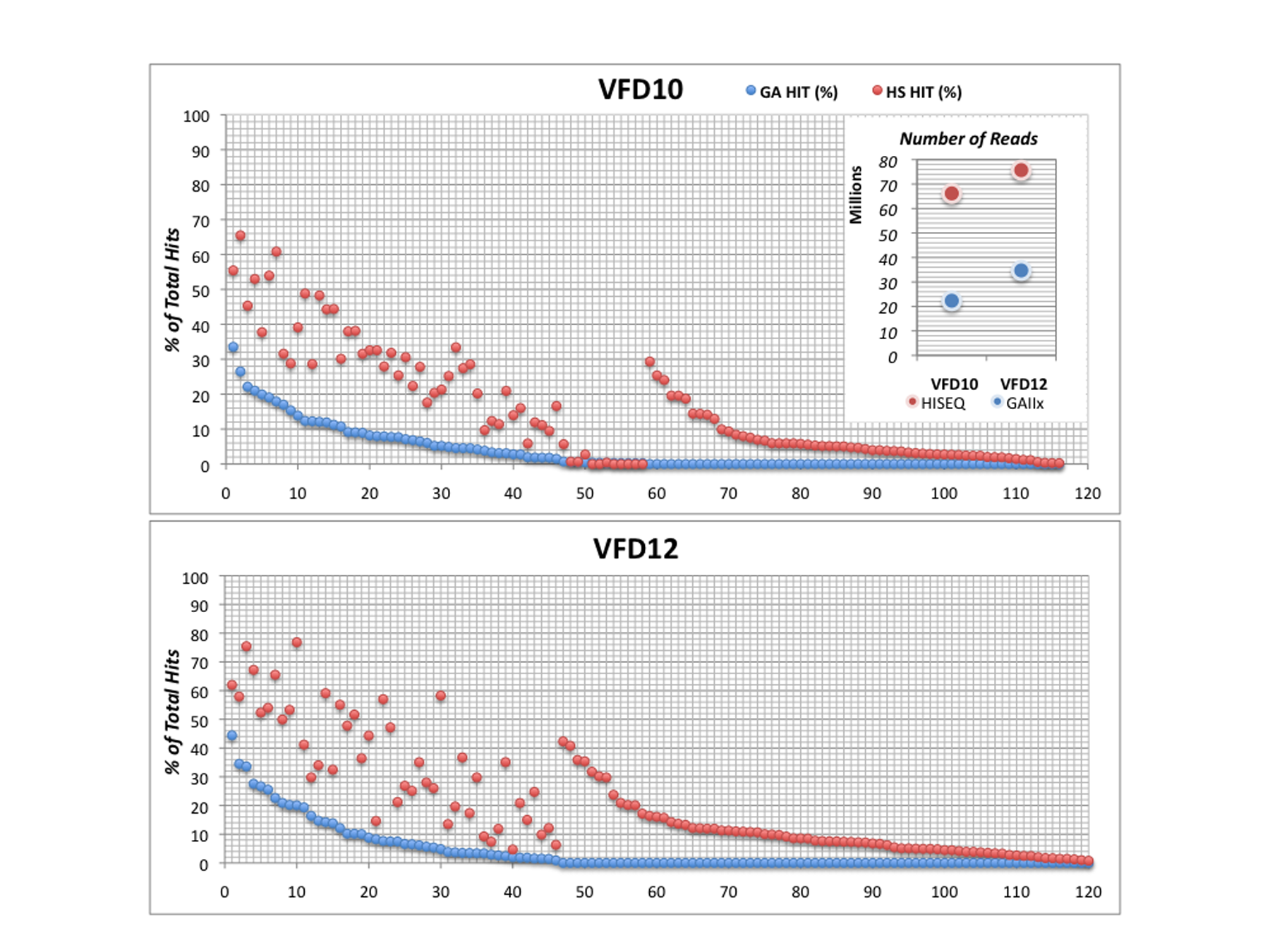

Supplement: Figure S4 — GENIUS 5VCE prediction of percent of total hits for identified bacterial species in VFD10 and VFD12, sequenced by both GAIIx and HiSeq 2000. The smaller chart to the upper right corner shows number of reads generated for two samples by GAIIx and HiSeq 2000. (TIFF) [file pone.0097699.s004.tiff]

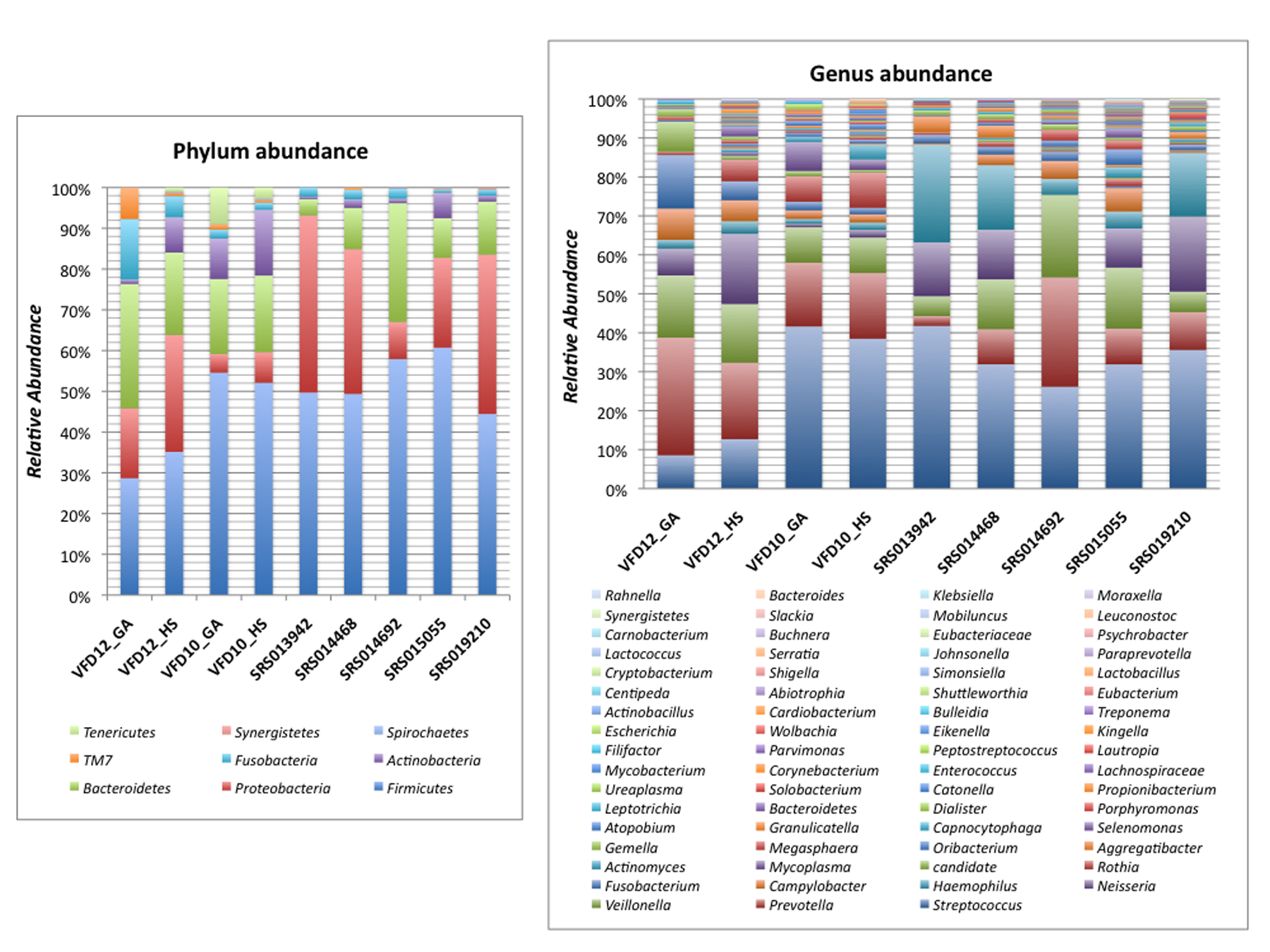

Supplement: Figure S5 — Abundance of bacterial phyla and genera identified in the salivary microbiome by GENIUS. (TIFF) [file pone.0097699.s005.tiff]

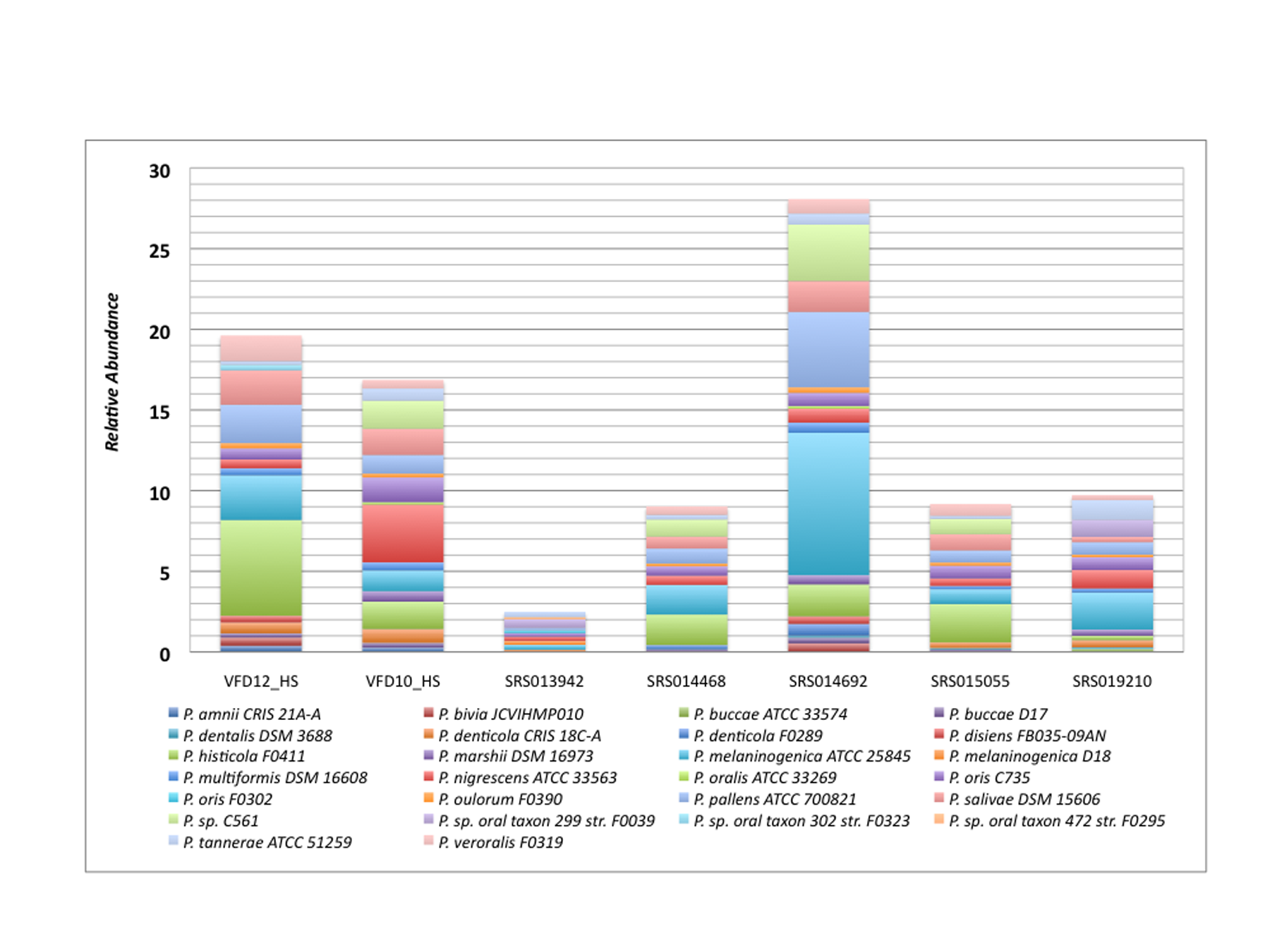

Supplement: Figure S6 — Distribution and abundance of different Prevotella spp. and strains in salivary microbiomes. (TIFF) [file pone.0097699.s006.tiff]

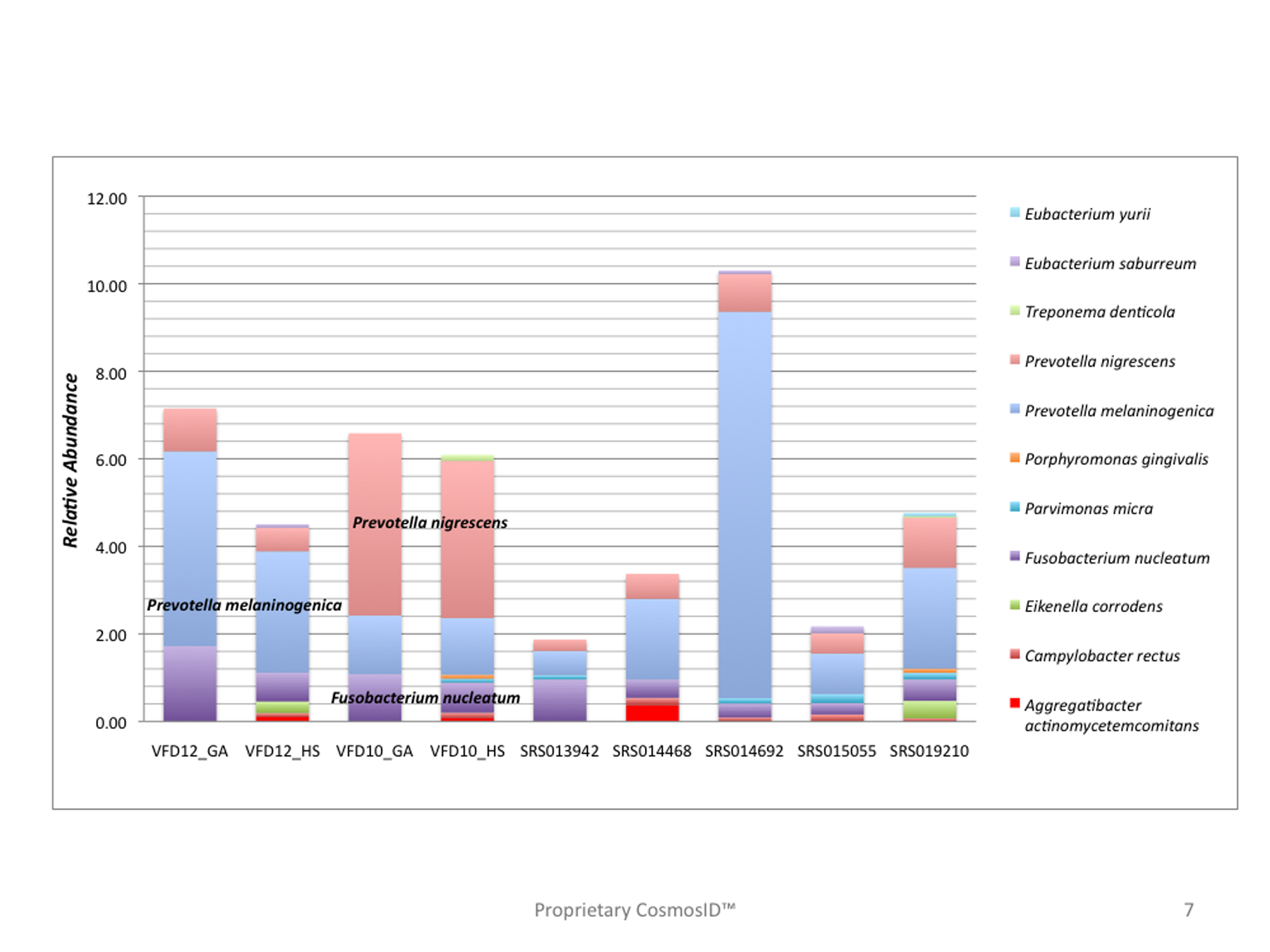

Supplement: Figure S7 — Occurrence and relative abundance of bacterial species associated with periodontal disease. (TIFF) [file pone.0097699.s007.tiff]

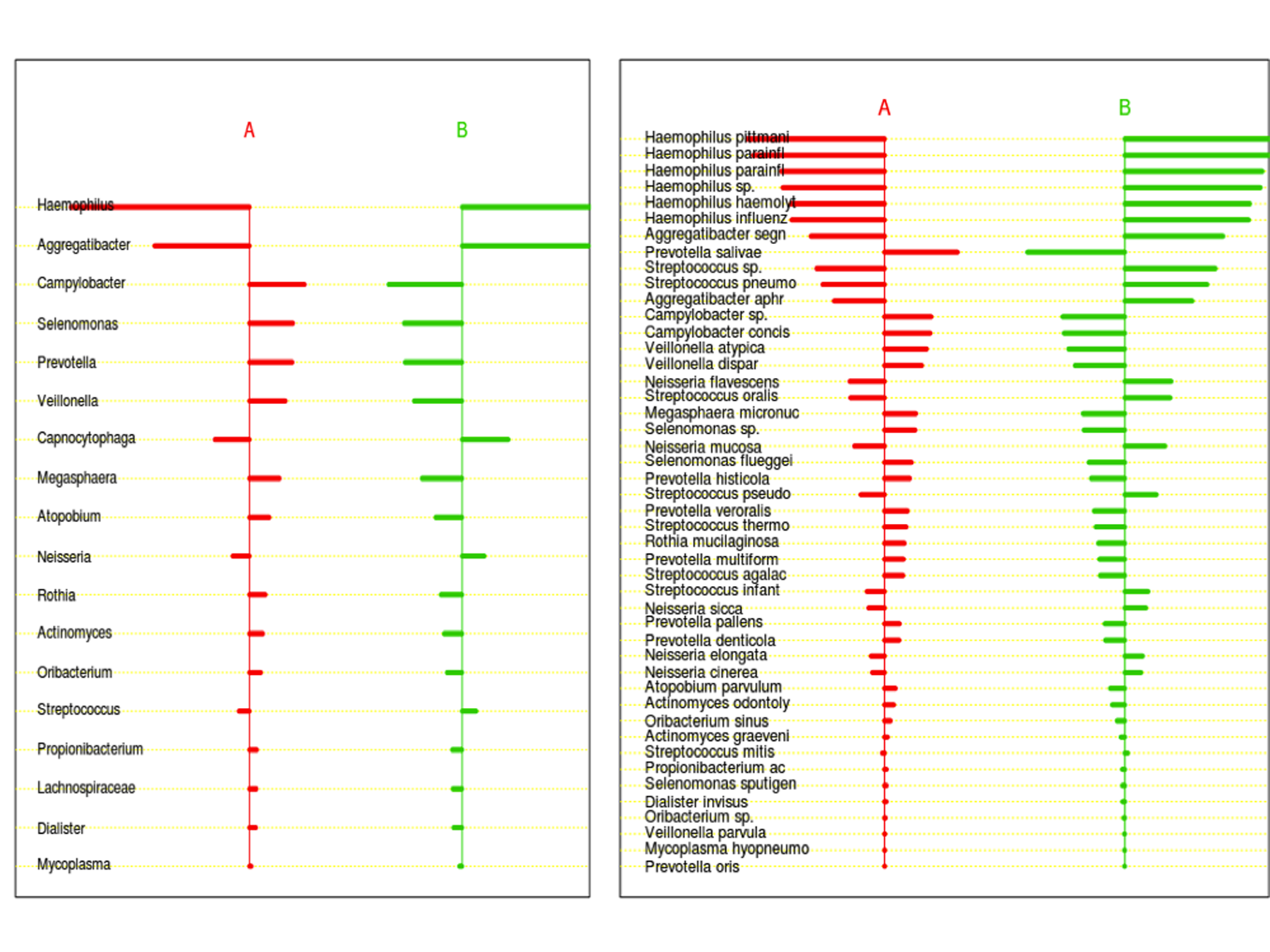

Supplement: Figure S8 — The centroid classification analysis of cluster A and B salivary samples. The top ranking markers can differentiate cluster B (green) and cluster A (red) by the centroid scores. Species and genera with nonzero components in each class are almost mutually exclusive. (TIFF) [file pone.0097699.s008.tiff]
