# Supplementary material for: Microbial Community Profiling of Human Saliva Using Shotgun Metagenomic Sequencing
Source: PLoS One. 2014 May 20;9(5):e97699. doi: 10.1371/journal.pone.0097699 (PMC4028220; doi:10.1371/journal.pone.0097699)
Supplement: Table S1 — Comparison of relative abundance of genera in VFD10-018 using different methods. (DOCX) [file pone.0097699.s009.docx]

**Table S1:** Comparison of relative abundance of genera (>1%) in VFD10-018 using three methods.

| **Genera** | **GAIIx GENIUS 5VCE** | **GAIIx GENIUS NmerCE** | **Ion Torrent NCBI 16S** | |
| --- | --- | --- | --- | --- |
| *Streptococcus* | 41.62% | 36.64% | 47.46% |  |
| *Prevotella* | 16.38% | 12.52% | 15.16% |  |
| *Veillonella* | 9.08% | 5.07% | 0.28% |  |
| *Rothia* | 6.54% | 3.72% | 1.87% |  |
| *Haemophilus* | 1.26% | 2.71% | 0.02% |  |
| *Campylobacter* | 2.20% | 1.35% | 0.00% |  |
| *Fusobacterium* | 2.21% | 2.71% | 8.75% |  |
| *Oribacterium* | 0.97% | 1.35% | 2.58% |  |
| unclassified Bacteroidetes | 0.87% | 1.35% | 0.00% |  |
| *Atopobium* | 1.24% | 3.04% | 3.88% |  |
| *Actinomyces* | 1.37% | 4.06% | 2.13% |  |
| *Megasphaera* | 0.67% | 1.35% | 3.04% |  |
| *Porphyromonas* | 0.56% | 1.35% | 0.56% |  |
| *unclassified Bacteria* | 1.35% | 2.37% | 0.00% |  |
| *Granulicatella* | 0.56% | 1.69% | 7.46% |  |
| *Neisseria* | 0.89% | 4.06% | 0.04% |  |
| *Capnocytophaga* | 0.60% | 4.06% | 0.62% |  |
| *Dialister* | 0.40% | 1.35% | 1.28% |  |
| *Solobacterium* | 0.27% | 1.35% | 0.10% |  |
| *Mycoplasma* | 7.47% | 0.00% | 0.05% |  |
| *Ureaplasma* | 1.48% | 0.00% | 0.00% |  |
| *Cardiobacterium* | 0.00% | 1.69% | 0.01% |  |
| *Filifactor* | 0.00% | 1.35% | 0.00% |  |
| *Gemella* | 0.00% | 1.35% | 0.31% |  |
| *Leptotrichia* | 0.00% | 1.35% | 0.12% |  |
| *Treponema* | 0.00% | 1.35% | 1.53% |  |
| Overall | 97.99% | 99.22% | 97.26% |  |
