# Supplementary material for: Microbial Community Profiling of Human Saliva Using Shotgun Metagenomic Sequencing
Source: PLoS One. 2014 May 20;9(5):e97699. doi: 10.1371/journal.pone.0097699 (PMC4028220; doi:10.1371/journal.pone.0097699)
Supplement: Table S2 — Comparison of relative abundance of genera in VFD12-006using different methods. (DOCX) [file pone.0097699.s010.docx]

**Table S2.** Comparison of relative abundance of bacterial genera (>1%) identified in VFD12-006 using three methods.

| **Genera** | **GAIIx GENIUS 5VCE** | **GAIIx GENIUS NmerCE** | **Ion Torrent NCBI 16S** |
| --- | --- | --- | --- |
| *Prevotella* |  | 24.24% | 28.35% |
| *Fusobacterium* | 13.73% | 8.02% | 31.86% |
| *Veillonella* | 15.96% | 7.39% | 1.60% |
| *Campylobacter* | 8.02% | 2.95% | 0.00% |
| *Neisseria* | 6.90% | 8.07% | 2.48% |
| Unclassified Bacteria | 7.70% | 3.67% | 0.00% |
| *Haemophilus* | 2.24% | 6.43% | 0.00% |
| *Streptococcus* | 8.55% | 14.23% | 6.91% |
| Rothia | 0.85% | 1.86% | 1.19% |
| *Megasphaera* | 0.89% | 1.86% | 3.72% |
| *Gemella* | 1.91% | 6.43% | 3.01% |
| *Leptotrichia* | 1.11% | 2.54% | 0.73% |
| *Selenomonas* | 0.28% | 0.00% | 5.38% |
| *Atopobium* | 0.24% | 1.40% | 0.68% |
| *Lachnospiraceae* | 0.22% | 1.95% | 1.06% |
| *Dialister* | 0.21% | 1.81% | 1.05% |
| *Granulicatella* | 0.22% | 1.54% | 1.25% |
| *Capnocytophaga* | 0.31% | 3.90% | 0.26% |
| *Corynebacterium* | 0.00% | 1.72% | 0.99% |
| *Porphyromonas* | 0.00% | 0.00% | 2.15% |
| *[Eubacterium] sulci* | 0.00% | 0.00% | 2.80% |
| *Actinobacillus* | 0.00% | 0.00% | 2.83% |
| Overall | 69.34% | 100.00% | 98.32% |
